# The interplay between molecular architecture, pharmacology, and suspected adverse drug reactions associated with nonsteroidal androgen antagonists in the United Kingdom

**DOI:** 10.1002/bcp.70569

**Published:** 2026-04-29

**Authors:** Simrit Dhillon, Albert A. Antolin, Alan M. Jones

**Affiliations:** ^1^ School of Pharmacy University of Birmingham Edgbaston UK; ^2^ ProCURE, Catalan Institute of Oncology (ICO) & Oncobell, Bellvitge Institute for Biomedical Research (IDIBELL) Barcelona Catalonia Spain; ^3^ Centre for Cancer Drug Discovery, Division of Cancer Therapeutics The Institute of Cancer Research London UK

**Keywords:** androgen receptor antagonist, apalutamide, bicalutamide, darolutamide, enzalutamide, pharmacovigilance, polypharmacology

## Abstract

**Aims:**

This work aimed to correlate potential links between the suspected adverse drug reaction (ADR) profile of licensed nonsteroidal androgen receptor antagonists (NSARA) with their unique chemical properties and known off‐target polypharmacology.

**Methods:**

Physicochemical and polypharmacology data were curated from the Electronic Medicines Compendium, FDA New Drug Applications documents, and ChEMBL database. Suspected ADRs and fatalities were curated from the United Kingdom Medicines and Healthcare products Regulatory Authority (MHRA) Yellow card spontaneous reporting scheme. The number of daily doses (*dd*) was extrapolated from OpenPrescribing and NHS Digital secondary care medicines data.

**Results:**

A total of *n* = 2522 suspected ADRs were associated with 42 903 000 *dd* of NSARAs usage in the United Kingdom. The highest number of ADRs were associated with enzalutamide (*n* = 1091) and bicalutamide (*n* = 738). Enzalutamide was found to have the most off‐target pharmacological interactions of the NSARAs studied (*n* = 4) including potent inhibition of γ‐aminobutyric acid, GABA receptor (IC_50_ = 2.6 μM *vs. C*_max_ = 7.7 μM and *C*_max, unbound_ = 185–212 nM) associated with nervous system disorders (*n* = 72, accounting for 67% of all NSARA ADRs in this SOC).

**Conclusions:**

Suspected skin and subcutaneous ADRs approached statistical significance and were interrogated for chemical and pharmacological connections for the first time with the aid of matched molecular pair (MMP) analysis. A potential correlation to nervous system disorders and cardiac arrhythmia for the GABA and *h*ERG inhibitors, enzalutamide and apalutamide, respectively was identified. Darolutamide's interaction with the 5‐HT (SERT) transporter may influence ADRs associated with cardiac and hepatobiliary SOCs.

**What is known about this subject**

- Nonsteroidal androgen receptor antagonists (NSARAs) are widely used in prostate cancer and are associated with heterogeneous adverse drug reaction (ADR) profiles.
- Off‐target pharmacology, including interactions with CNS and cardiac ion channels, has been proposed to contribute to NSARA‐related adverse effects.
- Spontaneous pharmacovigilance data can identify suspected ADR patterns, but links to molecular and pharmacological properties remain incompletely defined.

**What this study adds**

- This study integrates UK prescribing data, pharmacovigilance reports, and molecular pharmacology to systematically compare ADR profiles across licensed NSARAs.
- Distinct ADR patterns were associated with specific off‐target interactions (e.g., GABA, *h*ERG, and 5‐HT transporters), supporting a mechanistic basis for observed clinical differences.
- The findings demonstrate the value of combining molecular architecture and national registry data to improve early signal detection and risk stratification in clinical practice.

## 1 INTRODUCTION

In the United Kingdom, 52 300 men per year are diagnosed with prostate cancer,^1^ making it the most common cancer with peak diagnosis between 70 and 74 years old thus warranting ongoing pharmacovigilance studies.^1^ Hormone therapy is recommended as the first‐line treatment strategy by the National Institute for Health and Care Excellence (NICE).^2^, ^3^ Androgen receptor antagonists (ARA) inhibit the exertion of androgen‐like testosterone and dihydrotestosterone (DHT) pharmacological effects by competitively inhibiting testosterone binding to ARs.^4^ Steroidal ARAs treat a range of conditions including sexual deviation and hot flushes with gonadorelin analogue therapy.^5^

Nonsteroidal ARAs (NSARAs) are specifically used for the treatment of prostate cancer. NSARAs target prostate cancer cells by inhibiting androgen activity at the receptor level, therefore, decreasing prostate cancer cell growth.^6^ NSARAs can be either first generation (bicalutamide, flutamide), which exclusively target AR translocation to the nucleus, or second generation (enzalutamide, apalutamide, darolutamide), which have further improvements on this mechanism of action (MoA).^7^, ^8^

Monitoring adverse drug reactions (ADRs) reported to the MHRA Yellow Card Scheme in the United Kingdom^9^ is important as one in 16 patients admitted to hospital are suspected to be experiencing an ADR.^10^ The ability to determine correlation of NSARAs ADRs to molecular structure and/or off‐target pharmacological factors is therefore timely.^11^, ^12^, ^13^, ^14^, ^15^, ^16^, ^17^, ^18^

## 2 METHODS

**FIGURE 1:**
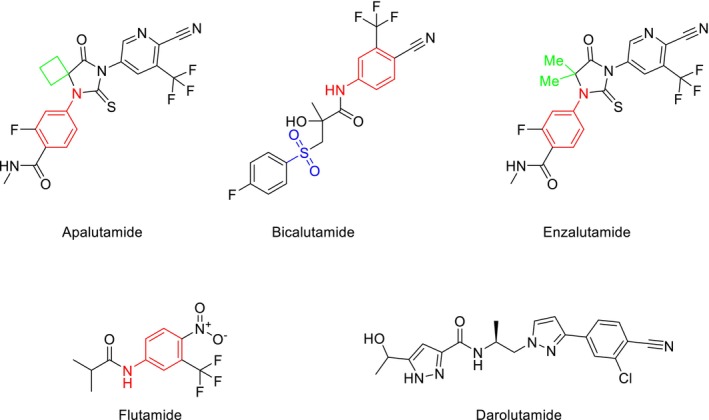
Structures of NSARAs studied. Colour coding: red for masked arylamine motif; blue for sulfonyl group; green for additional MMP analysis.

The licensed first generation NSARAs are bicalutamide^19^ and flutamide^20^; and second generation, enzalutamide,^21^ apalutamide,^22^ darolutamide^23^ (Figure 1) were selected based on inclusion and exclusion criteria of this study (Table 1).

**TABLE 1:** Inclusion and exclusion criteria.

Inclusion criteria	Exclusion criteria
ARA type:	ARA type:
Licensed nonsteroidal ARA (AR antagonists, first and second generation)	Steroidal ARA and nonsteroidal ARA–androgen synthesis inhibitors.
	Non licensed nonsteroidal ARA (AR antagonist) in the UK
Prescribing data in the UK (primary care and secondary care).	Nonsteroidal ARA with no prescribing data in the UK.
Available yellow card ADR reporting	No ADR reports on Yellow Card Scheme
Known age ranges for ADR reports.	Unknown age reports.

### 2.1 Prescribing data

NSARA data prescribing information was curated from OpenPrescribing database (for primary care prescribing^24^) and NHS Digital Secondary Care Medicines Data (SCMD).^25^ These datasets provided the tablet quantities of the NSARAs dispensed from each NHS trust and correspond to the licensing date for each NSARA studied (August 2018–December 2023).

### 2.2 Drug pharmacology

NSARA's molecular and physiochemical properties were extracted from the Chemical database of bioactive molecules with drug‐like properties, European Molecular Biology Laboratory (ChEMBL),^26^ FDA New Drug Application (NDA) documents,^27^, ^28^, ^29^, ^30^ and the Electronic Medicines Compendium (EMC).^31^ Dosing regimens were extracted from the British National Formulary (BNF).^32^

The following parameters were calculated: pIC_50_ was calculated from the negative log_10_ of the average AR IC_50_ data. Lipophilic ligand efficiency (LLE) was calculated using the following equation: pIC_50_ − log_10_P.^33^ The predicted blood–brain barrier (BBB) penetration requirements were interrogated as follows: molecular weight (MW) < 450 Da, <6 hydrogen bond donors (HBD), <2 hydrogen bond acceptor (HBA), a neutral or basic drug (indicated by pK_a_), topological polar surface area (*^t^* PSA) < 90 Å, log_10_D (at pH 7.4) between 1–3 and a low p‐glycoprotein affinity. The *C*_max_ was converted from ng/mL to nM based on the unique molecular weight of each NSARA.

### 2.3 Adverse drug reaction reporting

Suspected ADRs of all United Kingdom licensed NSARAs were extracted from the MHRA Yellow Card interactive drug analysis profile (iDAPs).^9^ Suspected ADRs reported for apalutamide, bicalutamide, enzalutamide, flutamide, and darolutamide (January 2018–December 2023 for bicalutamide; January 2018–October 2023 for the others) were extracted in accordance with the longitudinal timeline of NSARA prescribing in the United Kingdom.

Standardized NSARA ADRs were determined by calculating the ADR incidence rate per 100 000 daily doses (*dd*) for each drug:

1. Total tablets quantity dispensed divided by the respective standard of NSARA daily number of tablets = total *dd*.
2. Scale factor: Total *dd* dispensed/100 000.
3. ADRs multiplied by the scale factor gave the ADRs per 100 000 *dd* for each NSARA studied.

### 2.4 Target affinity

ChEMBL was used to extract the target proteins for each NSARA (accessed 29th November 2023).^26^ Where >1 IC_50_ for a human protein target was available, the mean IC_50_ value was calculated.

### 2.5 Ethical approval

Was not required by the School of Pharmacy sub‐ethics committee due to the use of fully anonymized patient data.

### 2.6 Statistical analysis

Excel for Microsoft 365 was used to perform chi‐squared (*χ* ^2^) tests on the standardized ADRs/100 000 *dd* data. A *p* < .05 was set for statistical significance.

### 2.7 Nomenclature of targets and ligands

Key protein targets and ligands in this article are hyperlinked to corresponding entries in http://www.guidetopharmacology.org and are permanently archived in the Concise Guide to PHARMACOLOGY 2023/24.^34^

## 3 RESULTS

### 3.1 Physicochemical properties and pharmacokinetics

Properties of the NSARAs (clog_10_P, pIC_50_, LLE) are shown in Table 2. Enzalutamide was the most lipophilic drug (clog_10_P = 3.99) and darolutamide was the most potent AR antagonist (pIC_50_ = 7.59). All AR antagonists had an LLE < 5. NSARAs that comply with the BBB criteria: bicalutamide, flutamide and darolutamide successfully passed the MW requirements; with apalutamide being the heaviest drug (477 Da). All the NSARA's failed the guidelines for number of HBAs by having >2 HBA's, however, passed the HBD criteria (<6). Apalutamide, enzalutamide, bicalutamide, and flutamide are neutral under clinical conditions. Apalutamide, enzalutamide and flutamide had a ^*t*^PSA < 90 Å. Bicalutamide and darolutamide met the criteria for log_10_D^7.4^ (2.88 and 2.67, respectively). Enzalutamide and flutamide were not P‐glycoprotein substrates. Overall, flutamide passed the most BBB requirements out of all AR antagonists (achieving 5/7 BBB requirements), whilst apalutamide met the least (3/7 conditions).

**TABLE 2:** Summary of the physicochemical, blood–brain barrier predicted penetration, and pharmacological properties of the five NSARAs.

Variable	Apalutamide		Bicalutamide		Enzalutamide		Flutamide		Darolutamide	
**Molecular obesity and on‐target efficiency metrics**										
clog_10_P	3.53		2.88		3.99		3.21		2.67	
AR pIC_50_	7.09		4.99		5.74		4.48		7.59	
LLE	3.58		2.11		1.75		1.27		4.92	
**Blood–brain barrier penetrant properties**										
MW (Da)	477.44		430.38		464.44		276.21		398.85	
pK_a_	13.05		11.78		13.05		12.81		9.81	
^*t*^PSA (Å)	89.33		107.26		76.44		72.24		119.62	
HB acceptors	5		4		4		3		4	
HB donors	1		2		1		1		3	
clog_10_D_7.4_	3.53		2.88		3.99		3.21		2.67	
P‐glycoprotein substrate	Yes		Yes		No		No		Yes	
No. of BBB requirements met	3		4		4		5		4	
**Pharmacokinetics**										
Bioavailability (F, %)	100		‐		84.2		‐		30	
*t*_½_ (h)	72		168		139		10		20	
*T*_max_ (h)	2		19	3	2		1.71	2.21	4	
*C*_max_ (nM)	2865 [A]	2817 [NDA]	1706 ((*R*)‐enantiomer)	195 ((*S*)‐enantiomer)	7710 [E]	5898 [NDE]	162 [F]	2643 [H]	530 [D]	21 419 [KD]
*C*_max, unbound_ (nM)	115	141	17	8	185	212	32	846	41	
F_u_ (%)	4	5	1	4	2.4	3.6	20	32	7.8	
Hepatic metabolism	Yes: CYP‐2C8, ‐2C9, ‐2C19, ‐3A4,	Yes: [C]	Yes: CYP3A4	Yes: Isoenzyme & glucuronidation	Yes: CYP‐2C8, ‐2C9, ‐2C19, ‐3A4,	Yes: [C]	Yes: CYP‐ 1A2	‐	Yes: CYP‐3A4	
Renal excretion (%)	65		50		71		45		63	
Volume of distribution (L)	276		n.r.		110		n.r.		119	n.r.
Clearance (L/h)	1.30		0.32		0.54		n.r.		6.96	
PPB (%)	96 [A]	95 [NDA]	99 ((R)‐enantiomer)	96% ((S)‐enantiomer)	97.5 [E]	95% [NDE]	95 [F]	93% [H]	92 [D]	99.8 [KD]
Dosing	OD (240 mg)		OD (50 mg) or OD (150 mg)		OD (160 mg)		TDS 3× (250 mg)		BD 2× (300 mg)	

*Note*: Colour coded green met the BBB thresholds.

Abbreviations: [A], apalutamide; [B], bicalutamide; BBB, blood–brain barrier; [C], carboxylesterases; clog_10_D7.4, calculated log_10_D at pH 7.4; clog_10_P, calculated log_10_P; *C*_max_, peak serum concentration; [D], darolutamide; [E], enzalutamide; [F], flutamide; *F*_u_, fraction unbound; HB, hydrogen bond; [HF], hydroxyflutamide; [KD], ketodarolutamide; LLE, lipophilic ligand efficiency; MW, molecular weight; [NDA], *N*‐desmethyl apalutamide; [NDE], *N*‐desmethyl enzalutamide; n.r., not reported; pKa, acid dissociation constant; PPB, plasma protein binding; *T*_max_, time taken to reach Cmax; ^*t*^PSA, total polar surface area.

In clinical settings, darolutamide did not significantly alter cerebral blood flow (CBF), consistent with its low clinical BBB penetration and subsequent low risk of CNS‐related adverse events.^35^ However, a significant reduction in CBF was observed with enzalutamide suggestive of increased BBB penetration.^35^ This was further supported by cerebrospinal fluid (CSF) for enzalutamide in rats showing a 3%–6% penetration.^36^ CSF measurement in dogs for apalutamide show a 2%–5% penetration,^37^ A single case report for CSF leak with flutamide was identified^38^ but no clinical data was identified for bicalutamide.

Flutamide required the most daily tablets; with the regime consisting of one tablet, three times a day due to the short half‐life (10 h). Apalutamide had the largest volume of distribution (276 L) with bicalutamide and flutamide having undetermined volumes of distribution (V_d_).

### 3.2 Target affinity

Table 3 shows the strength of inhibition assessed by using the mean IC_50_ values. Apalutamide and darolutamide had the most potent inhibition of the AR receptors, with IC_50_ = 63 and 26 nM, respectively. Bicalutamide and flutamide had the weakest AR antagonist inhibition (1.2 and 1.3 μM, respectively). Bicalutamide had additional interactions with progesterone receptors at clinically achievable levels (IC_50_ = 1.8 μM *vs. C*_max_ = 1.7 μM). Enzalutamide was found to have the most off‐target pharmacological interactions of the NSARAs studied (*n* = 5) including potent inhibition of γ‐aminobutyric acid, GABA‐α receptor (IC_50_ = 2.6 μM *vs. C*_max_ = 7.7 μM and unbound *C*_max_ = 185–212 nM). Apalutamide, was the only other GABA (unspecified α/β subtype) inhibitor (IC_50_ = 3 μM *vs. C*_max_ = 2.9 μM) and a modest inhibitor of the human Ether‐à‐go‐go‐Related Gene (hERG) ion channel (IC_50_ = 6 μM *vs. C*_max_ = 2.9 μM and unbound *C*_max_ = 115–141 nM). Enzalutamide was a significantly weaker *h*ERG inhibitor (15.7 μM *vs. C*_max_ = 7.7 μM). Furthermore, darolutamide was the only NSARA to show effects at the SERT transporter at <10 μM.

**TABLE 3:** Target pharmacology (mean IC_50_ (nM)) of the five NSARAs studied alongside *C*_max_ and clinical measurements for BBB penetration. N.R. = not reported.

	Apalutamide	Bicalutamide	Enzalutamide	Flutamide	Darolutamide
AR	63	1167	806	1348	26
Glucocorticoid receptor	‐	‐	<19 500	‐	‐
Progesterone receptor	‐	1819	‐	‐	‐
Bile salts export pump	‐	54 450	‐	79 396	‐
Progesterone receptor	No inhibition	‐	16 000	‐	‐
GABA	3000	‐	2600	‐	‐
*h*ERG	6000	‐	15 700	‐	‐
5‐HT transporter	‐	‐	‐	‐	<10 000
*C*_max_	**2865**	**1706**	**7710**	**162**	**530**
*C*_max, unbound_	115–141	8–17	185–212	32–846	41
BBB penetration	2%–5% (dog)^37^	n.r.	3%–6% (rat)^36^	CSF leak case report^38^	0.3%–0.6% (human)^35^
	10–100 nM				
	100–1000 nM				
	1000–10 000 nM				
	>10 000 nM				
‐	Undetermined				

### 3.3 Descriptive total ADRs and fatalities since drug launch date

**FIGURE 2:**
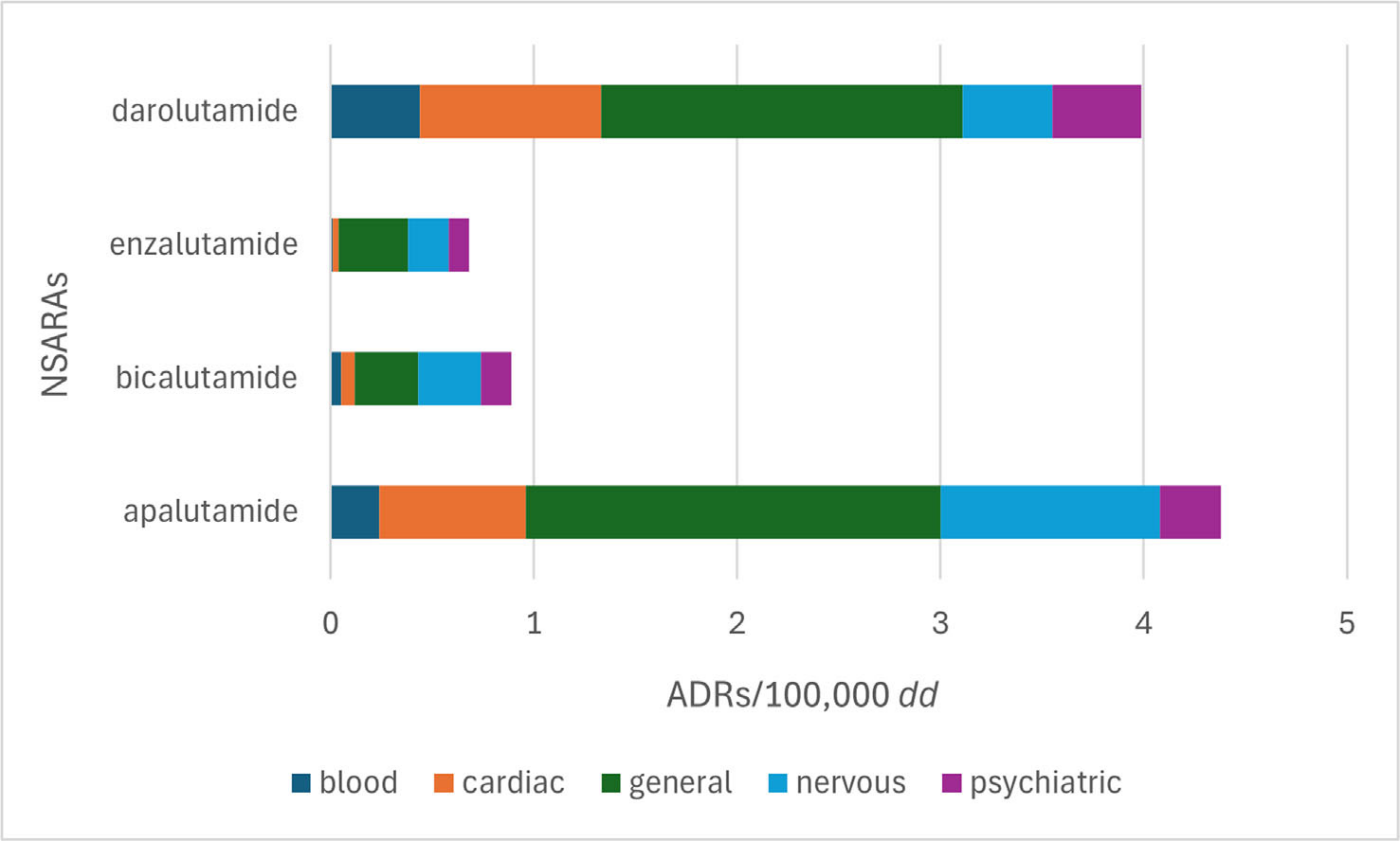
Bar chart comparison of the ADRs per 100 000 *dd* for the main SOC areas excluding flutamide (*n* = 1 ADR in these seven major SOCs).

ADR profiles of the NSARAs were standardized based on the number of *dd* dispensed in the time period of the study (Table 4). Overall, enzalutamide had the largest number of suspected ADRs, followed by bicalutamide (738), with darolutamide the least (98). Enzalutamide had the largest total number of fatalities (*n* = 54). Flutamide had the next highest at 24. Darolutamide was the only NSARA to have no reported deaths in this time period (Figure 2). Flutamide had no ADRs during the time period of this study and is excluded from further discussion.

**TABLE 4:** Summary of the suspected ADRs for the five NSARAs. Key: Total ADRs calculated by total ADRs/100 000 dd; fatalities calculated by fatalities/100 000 dd; *values* calculated by chi‐squared test; *p* values exclude flutamide and where there are *n* < 3 reports.

	Apalutamide	Bicalutamide	Enzalutamide	Flutamide	Darolutamide	*P*
Number of *dd* (/100 000)	1 700 000 (17.0)	5 500 000 (55.0)	35 500 000 (355.0)	3000 (0.03)	200 000 (2.0)	‐
Total ADRs						
Since launch to 2023	206	738	1081	399	98	
2018–2023	206	155	608	0	98	
Fatalities						
Since launch to 2023	3	22	54	24	0	
2018–2023	3	0	35	0	0	
**Blood and lymphatic system disorders**						
Total ADRs	4 (0.24)	3 (0.05)	4 (0.01)	0	1 (0.44)	0.89
Fatalities	0	0	0	0	0	‐
Thrombocytopenia	2 (0.12)	0	1 (0.002)	0	0	‐
Neutropenia	0	0	1 (0.002)	0	1 (0.44)	‐
**Cardiac disorders**						
Total ADRs	12 (0.72)	4 (0.07)	12 (0.03)	0	2 (0.89)	0.70
Fatalities	1 (0.06)	0	0	0	0	‐
Cardiac Arrhythmia's	5 (0.30)	0	4 (0.01)	0	0	‐
Heart failure	6 (0.36)	0	0	0	1 (0.44)	‐
**General disorders and administration site conditions**						
Total ADRs	34 (2.04)	17 (0.31)	121 (0.34)	0	4 (1.78)	0.52
Fatalities	1 (0.06)	0	29 (0.08)	0	0	‐
**Nervous system disorders**						
Total ADRs	18 (1.08)	17 (0.31)	72 (0.20)	0	1 (0.44)	0.82
Fatalities	0	0	0	0	0	‐
Neurological disorders NEC	8 (0.48)	8 (0.15)	33 (0.09)	0	1 (0.44)	
**Psychiatric disorders**						
Total ADRs	5 (0.30)	8 (0.15)	37 (0.10)	0	1 (0.44)	0.94
Fatalities	0	0	0	0	0	‐
**Hepatobiliary disorder**						
Total ADRs	0	5 (0.09)	2 (0.01)	0	1 (0.44)	‐
Fatalities	0	0	0	0	0	
**Skin and subcutaneous tissue disorders**						
Total ADRs	40 (2.40)	18 (0.33)	16 (0.05)	0	6 (2.67)	0.25
Fatalities	0	0	0	0	0	‐
Epidermal	36 (2.16	15 (0.27)	12 (0.03	0	6 (2.67)	0.25

#### 3.3.1 Blood and lymphatic system disorder ADRs and fatalities

Darolutamide and apalutamide had the highest reports of 0.44 and 0.24 ADRs per 100 000 *dd*, respectively. Patients taking darolutamide experienced suspected neutropenia (0.44/100 000 *dd*) and apalutamide experienced suspected thrombocytopenia (0.12/100 000 *dd*).

#### 3.3.2 Cardiac disorders ADRs and fatalities

Darolutamide had the highest suspected cardiac disorder profile (0.89/100 000 *dd*), apalutamide had the second highest ADRs/100 000 *dd* (0.72), along with being the only AR antagonist to have a suspected fatality in this category. The main cardiac event possessed by apalutamide patients were cardiac arrhythmias and heart failure, 0.30 and 0.36 per 100 000 *dd*, respectively. Whilst enzalutamide exhibited the least cardiac events (0.03 per 100 000 *dd*).

#### 3.3.3 General disorders and administration site conditions ADRs and fatalities

General disorders and administration site conditions had the highest number of ADRs across all AR antagonists. Enzalutamide had a fatality rate of 0.08 per 100 000 *dd*. Apalutamide also experienced a high prevalence of general disorders side effects (2.04/100000 *dd*) and fatalities (0.06/100000 *dd*). Darolutamide followed with 1.78 ADRs/100000 *dd*.

#### 3.3.4 Nervous system ADRs and fatalities

Similarly, to general disorder ADRs, apalutamide (1.08/100000 *dd*) and darolutamide (0.44/100000 *dd*) had the highest nervous system ADRs. Apalutamide's main nervous system ADR was neurological disorders (0.48/100000 *dd*).

#### 3.3.5 Psychiatric disorders ADRs and fatalities

Darolutamide had 0.44 ADRs per 100 000 *dd*, which was the highest number of suspected events for the NSARA series. Patients taking enzalutamide experienced 0.10 suspected psychiatric ADRs/100 000 *dd*.

#### 3.3.6 Hepatobiliary disorders ADRs and fatalities

The highest suspected ADRs of patients with hepatobiliary disorders were experienced with darolutamide (0.44 per 100 000 *dd*) but may be an artefact of the very low number of *ADRs* (*n* = 1).

#### 3.3.7 Skin and subcutaneous tissue disorder ADRs and fatalities

Similar to general disorders, skin and subcutaneous disorders had one of the largest numbers of suspected ADRs/100 000 *dd*. Darolutamide (2.67/100 000 *dd*) and apalutamide (2.40/100 000 *dd*) had several ADRs, with epidermal ADRs predominating in this category. All darolutamide ADRs being epidermal and 2.16 ADR's/100 000 *dd* for apalutamide.

#### 3.3.8 Age range of NSARA ADR SOC reports

Apalutamide consisted of 72 patients who experienced a suspected ADR between the ages of 50–89 years old. Bicalutamide had 2 patients between 0 and 19 years old, with the remaining 35 patients between 60 and 99 years old. Enzalutamide consisted of 140 patients between 50 and 99 years old and darolutamide having 14 patients between the ages of 60 and 89.

Molecular matched pair (MMP) analysis was possible between apalutamide versus enzalutamide (Figure 1) due to a single point variation present—*gem*‐dimethyl versus *spiro*cyclobutane, respectively—on the thiohydantoin scaffold. This led to a logarithmic difference in activity at the AR (63 *vs*. 806 nM), and a 2.5‐fold difference in *h*ERG activity (6000 *vs*. 15 700 nM), comparable inhibition at the GABAα/GABA unspecified receptor (enzalutamide and apalutamide, respectively), and no reported inhibition at glucocorticoid and progesterone receptors (GR/PR) for apalutamide and sole weak GR inhibition for enzalutamide, respectively.

## 4 DISCUSSION

### 4.1 Total ADRs

The highest number of total ADRs reported in the 5‐year time period was with enzalutamide (*n* = 1091), followed by bicalutamide (*n* = 738) and darolutamide (*n* = 35).

Flutamide's data were excluded when comparing individual SOC ADRs per 100 000 *dd* as there was limited prescribing during the time frame of this study (3000 *dd*) versus all other NSARAs 42 900 000 *dd* and no ADRs reported in these specific SOC classes.

### 4.2 Total fatalities

Enzalutamide emerged as having the highest number of fatalities (*n* = 54). Flutamide had zero reported fatalities during the time period of this study.

### 4.3 Blood and lymphatic systems disorders ADRs and fatalities

Darolutamide had a prevalence of 0.44 ADRs/100 000 *dd* with one report of neutropenia. Apalutamide followed with 0.24 ADRs/100 000 *dd*, with two reports of thrombocytopenia. Interactions between apalutamide and CYP2C8 and CYP2C9 can lead to drug–drug interactions (DDI). Enzalutamide patients experienced both immune thrombocytopenia and neutropenia again possibly due to DDIs with CYP2C8 and CYP2C9.

### 4.4 Cardiac disorders ADRs and fatalities

Darolutamide had the greatest prevalence of ADRs for cardiac disorders (0.89 ADRs/100 000 *R*_*x*_). The increase in cardiovascular events on darolutamide could be potentially ascribed to SERT inhibition, given its importance in the cardiovascular system.^39^ Furthermore, darolutamide is a known CYP3A4 inhibitor related to potential DDIs. Prostate cancer patients are within the same age group to have cardiac co‐morbidities. Cardiac medications are known CYP3A4 substrates (e.g., simvastatin/atorvastatin^40^). Additionally, p‐gp modulation causes disturbances in ion efflux, which can be of concern to those taking ion channel inhibitors, such as verapamil and amiodarone.^41^ P‐glycoprotein inhibitors include proton‐pump inhibitors (e.g., omeprazole) and macrolides antibiotics (e.g., clarithromycin and erythromycin). The combination of these factors may have a detrimental impact on the patients' health by increasing the risk for a cardiovascular event due to polypharmacy in this age group.

Apalutamide was a modest inhibitor of the human Ether‐à‐go‐go‐Related Gene (*h*ERG) ion channel (IC_50_ = 6000 nM *vs. C*_max_ = 2900 nM / unbound = 115–141 nM) and had the highest rate of suspected cardiac arrhythmia ADRs, 30‐fold over the only other NSARA with this suspicion, enzalutamide, a significantly weaker *h*ERG inhibitor (15 700 nM *vs. C*_max_ = 7700 nM/unbound 185–212 nM).

Apalutamide (0.36 ADRs/100 000 *dd*) also interacts with CYP2C8, CYP2C9, CYP2C19 and P‐gp. Inducing CYP2C19 can cause patients taking clopidogrel to increase metabolism to its active metabolite, which has a half‐life of approx. 30 min. This causes the therapeutic effect to diminish quickly after administration, making the patient at risk for an ischemic attack dependent on polypharmacy confounders not available in these datasets.

Enzalutamide is also known to interact with CYP2C8, CYP2C9, CYP2C19, CYP3A4. Bicalutamide is a CYP3A4 inhibitor and p‐gp inducer, cardiac events are potentially due to DDIs.

### 4.5 General disorders and administration site conditions ADRs and fatalities

A higher prevalence of ADRs was observed with enzalutamide, apalutamide and darolutamide for general disorders and administration site conditions. Apalutamide's interaction with CYP3A4, CYP2C19 and p‐gp can induce DDI with other drugs that utilize their metabolic pathways. Darolutamide and bicalutamide affects CYP3A4 and p‐gp, whilst enzalutamide interacts with CYP3A4. Enzalutamide had 0.08 fatalities/100 000 *dd*, followed by apalutamide (0.06).

### 4.6 Nervous system ADRs and fatalities

Effects of ARs on CNS and cognitive function have been reported.^42^ Apalutamide and darolutamide had 1.08 and 0.44 nervous system ADRs/100 000 *dd*, respectively. These higher reports are potentially due to their unique pharmacology. Inhibition of GABA receptor can lead to increased anxiety, stress, and fear responses and apalutamide, a GABA inhibitor (IC_50_ = 3 μM *vs. C*_max_ = 2.9 μM) had the highest relative rate of suspected nervous system ADRs at 1.08 per 100 000 *dd*. In clinical settings, darolutamide did not significantly alter cerebral blood flow (CBF), consistent with its low clinical and predicted BBB penetration and subsequent low risk of CNS‐related adverse events.^35^ In contrast, CSF measurement in dogs for apalutamide show a 2%–5% penetration, which may inform patient stratification.^37^

### 4.7 Psychiatric disorder ADRs and fatalities

Darolutamide and apalutamide led with the highest number of psychiatric disorders per 100 000 *dd* (0.44 and 0.33, respectively). Darolutamide was identified as the sole NSARA to show effects at 5‐HT (serotonin) transporter at <10 μM. 5‐HT has a well‐known role in depression, obsessive‐compulsive disorder, and social phobia and there have been reports of increased depression in patients taking AR antagonists.^43^ However, low BBB penetration of darolutamide made this off‐target interaction an unlikely contributor to suspected psychiatric disorders reported. Similar to the nervous system side effect profile bicalutamide and enzalutamide followed with lower numbers of suspected reports, 0.15 and 0.1 ADRs per 100 000 *dd*.

### 4.8 Hepatobiliary disorders ADRs

Bicalutamide and flutamide both weakly inhibited BSEP. Chronic inhibition of BSEP can result in cholestasis due to the restriction of the bile passage.^44^

Darolutamide followed with 0.44 ADRs/100 000 *dd*. Darolutamide was the only NSARA to show effects were 5‐HT (serotonin) transporter at <10 μM and an association of inhibiting 5‐HT and dysregulation of crucial components of liver function including hepatic blood flow, innervation and wound healing was noted.^45^

Apalutamide and enzalutamide did not reveal significant reports of hepatic injuries, due to possessing similar drug chemical properties. They are cleared *via* the kidneys with a renal excretion of 65% and 71%, respectively.

### 4.9 Skin and subcutaneous tissue disorder ADRs and fatalities

NSARAs exhibited suspected skin and subcutaneous tissue disorder ADRs impacting the epidermis. Apalutamide, darolutamide and bicalutamide possessed the largest number of ADRs/100 000 *dd* (2.40, 2.67, and 0.33, respectively). This may be due to the apalutamide containing an arylamine (Figure 1), which are molecular characteristics known to induce skin reactions.^46^ Conversely, flutamide also contains an arylamine, but there were no reports of suspected skin or subcutaneous tissue ADRs. Bicalutamide (Figure 1) contains a sulfonyl‐arylamine, which is known to induce inflammation via IgE and IgG immune pathways.^47^ Resulting in patients with a sulfonyl‐arylamine allergy that are taking bicalutamide to experience epidermal side effects due to hypersensitivity as a potential cause.

## 5 LIMITATIONS

Reports from the Yellow Card scheme are suspected reports, and therefore, no causal relationship must be proved before submitting a report. Therefore, reported ADRs in any registry may have no defined relationship to the pharmacology of the NSARAs. Under‐reporting of ADRs is also a common issue with pharmacovigilance schemes typically estimated to be 6% of all ADRs.^48^ Independent datasets were used for frequency of dispensed prescriptions and frequency of reported suspected ADRs; neither dataset enables the interpretation of co‐morbidity and co‐prescription confounders, for example. This limits the assessment of causality and identification and adjustment for these confounding factors. Prostate cancer occurs in later stages of life where issues of polypharmacy medications for co‐morbidities can arise.

Furthermore, the prescriber's choice of a NSARA may be influenced by known interactions (and contraindications) with drugs co‐prescribed for co‐morbidities, i.e., there may be a significant bias by indication (or contraindication) as these comorbidities may affect sensitivity for ADRs. For example, certain NSARAs are currently cautioned as follows; enzalutamide: seizures, encephalopathy, hypersensitivity, ischemic heart disease, falls and embryo‐foetal tox; darolutamide: seizures, ischemic heart disease and embryo‐foetal tox; and apalutamide: seizures, ischemic heart disease, fractures, falls and embryo‐foetal tox. The study cannot adjust for these confounders. There is also a NSARA dose *vs*. risk of association with an ADR that cannot be described within these datasets.

In this study, we did not apply structured tools such as the Liverpool Causality Assessment Tool (LCAT)^49^ or the Naranjo Scale,^50^ as the nature of Yellow Card reports limits the feasibility of such methods. The MHRA Yellow Card dataset contains spontaneously reported, anonymized suspected ADRs, and typically lacks the detailed temporal, clinical, laboratory, and de challenge/re challenge information required to reliably score cases using either instrument. As a result, there was insufficient granularity within the available data to apply LCAT or the Naranjo algorithm in a meaningful or reproducible way.

### 5.1 Implications for future research and clinical practice

The findings of this study suggest several avenues for further investigation. First, the correlations identified between specific polypharmacological features (e.g., GABA or *h*ERG inhibition, 5 HT interactions) and suspected ADR patterns support the need for mechanistic studies and controlled clinical cohorts to test the causality of these associations. Second, the substantial variability in suspected ADR incidence across NSARAs highlights the potential value of stratifying patients by co‐morbidities, polypharmacy, and predicted blood–brain barrier penetration in future pharmacovigilance or pharmacoepidemiologic work. Clinically, improved understanding of off‐target interactions may assist prescribers in selecting NSARAs based on individual patient risk factors—particularly in older populations with cardiovascular, neurological, or hepatic vulnerabilities. Finally, this approach demonstrates the utility of integrating national prescribing, pharmacology, and ADR datasets to support early signal detection, which could be extended to other endocrine oncology drug classes.

## 6 CONCLUSIONS

The detection of suspected ADRs of NSARAs and potential correlations to their respected polypharmacology and physicochemical properties was identified. The highest number of ADRs were associated with enzalutamide (*n* = 1091) and bicalutamide (*n* = 738). Enzalutamide was found to have the most off‐target pharmacological interactions of the NSARAs studied (*n* = 4) including potent inhibition of GABAα receptor (IC_50_ = 2.6 μM *vs. C*_max_ = 7.7 μM and *C*_max,u_ = 185–212 nM) and had the most ADR reports for nervous system disorders (*n* = 72, accounting for 67% of all NSARA ADRs in this SOC). Apalutamide, the only other GABA (unspecified α/β) inhibitor (IC_50_ = 3000 nM *vs. C*_max_ = 2900 nM and *C*_max,u_ = 115–141 nM) had the next highest number of reports of suspected nervous system ADRs at 18 cases.

Apalutamide was a modest inhibitor of the *h*ERG ion channel (IC_50_ = 6000 nM *vs. C*_max_ = 2900 nM and *C*_max,u_ = 115–141 nM) and had the highest rate of suspected cardiac arrhythmia ADRs, 30‐fold over the only other NSARA with this suspicion, enzalutamide, a significantly weaker *h*ERG inhibitor (15 700 nM *vs. C*_max_ = 7700 nM and *C*_max,u_ = 185–212 nM). Darolutamide was the only NSARA to show effects at the SERT transporter at <10 μM but did not translate to a correlation to psychiatric disorders due to low BBB penetration but may be associated with both cardiac and hepatobiliary suspected ADRs reported.

## AUTHOR CONTRIBUTIONS

S.D. carried out the data acquisition, analysis and interpretation, and drafted and revised the manuscript. A.A.A. carried out data analysis and interpretation, and revised the manuscript. A.M.J. conceived and designed the study including analysis and interpretation of data for the work, and supervised, drafted and revised the manuscript. All authors gave final approval to the version to be published and agree to be accountable for all aspects of the work in ensuring that questions related to the accuracy or integrity of any part of the work are appropriately investigated and resolved.

## CONFLICT OF INTEREST STATEMENT

A.A.A. is an ISCIII–Miguel Servet Fellow supported by the Instituto de Salut Carlos III grant CP23/00115 and by the Spanish Ministry of Science and Innovation (MCIN/AEI) [PID2022‐136344OA‐I00]; CERCA Program/Generalitat de Catalunya, and FEDER funds/European Regional Development Fund (ERDF)–a way to build Europe. A.A.A. is an employee of the Vall d'Hebron Institute of Oncology (VHIO) and A.A.A. and A.M.J. were previously employees of The Institute of Cancer Research (ICR), both of which have commercial interests in a range of drug targets and therapeutics. VHIO and the ICR operate a Rewards to Discoverers scheme whereby their employees may receive financial benefits following the commercial licensing of a project. A.A.A. is/was a consultant to Darwin Health and has received grant funding from Vivan Therapeutics and ATG Therapeutics.

## Data Availability

The data that supports the findings of this study are available within this article. The datasets analysed during the current study are available in the interactive drug analysis profiles, MHRA, Yellow Card repository: https://yellowcard.mhra.gov.uk/idaps.
